# The Collins Case

**Published:** 1912-02

**Authors:** 


### Abstracts and Selections.

### The Collins Case.

The case of Collins vs. the State of Texas, involving the constitutionality of the one board medical act, of the Thirtieth Legislature, will come before the Supreme Court of the United States Friday, the 19th, and Attorney General Jewel P. Lightfoot will leave today for Washington to represent the State in this case. The Court of Criminal Appeals upheld the constitutionality of the act and appeal was taken directly to the Supreme Court of the United States.

The case first came to the Court of Criminal Appeals in habeas corpus proceedings, and the relator, an El Paso osteopath, was remanded. The charge against Collins was that he violated the one board medical act by practicing osteopathy without having obtained a verification license from the State Board of Medical Examiners.

Collins now contends that he is deprived of his rights as a citizen of the United States by being deprived of the privilege to practice under his diploma as he did before the one board medical act became effective. Likewise the plea is made that his privileges and immunities are affected under the Fourteenth Amendment to the Constitution, in that his property rights in his diploma are injured. He insists that previously he was not required to stand examination, and that the act in question discriminates between osteopaths and the general practice of masseurs, trained nurses and druggists, who supply medicines on request for a remedy for certain ailments, while not being permitted to prescribe, etc.

The relator is a graduate of the American School of Osteopathy at Kirksville, Mo., and began practice at El Paso in 1903 on his diploma received from the parent school. The one board medical act was passed in 1907 and required all persons practicing medicine and assuming to cure diseases, either to present within one year their diplomas from some reputable school or old licenses to the board for the purpose of securing a verification license, or to stand an examination before the board and secure a license by this means. Collins did not get his verification license, either through neglect or disinclination to do so, but wished to continue to practice under his old diploma. Hence proceedings were instituted against him, and have now gone to the United States Supreme Court.—*Statesman.*

